# Histone Demethylases ELF6 and JMJ13 Antagonistically Regulate Self-Fertility in Arabidopsis

**DOI:** 10.3389/fpls.2021.640135

**Published:** 2021-02-12

**Authors:** Charlie Keyzor, Benoit Mermaz, Efstathios Trigazis, SoYoung Jo, Jie Song

**Affiliations:** Department of Life Sciences, Imperial College London, London, United Kingdom

**Keywords:** chromatin regulation, histone modification, histone demethylases, epigenetics, flower development, self-fertility

## Abstract

The chromatin modification H3K27me3 is involved in almost every developmental stage in Arabidopsis. Much remains unknown about the dynamic regulation of this histone modification in flower development and control of self-fertility. Here we demonstrate that the H3K27me3-specific demethylases ELF6 and JMJ13 antagonistically regulate carpel and stamen growth and thus modulate self-fertility. Transcriptome and epigenome data are used to identify potential targets of ELF6 and JMJ13 responsible for these physiological functions. We find that ELF6 relieves expansin genes of epigenetic silencing to promote cell elongation in the carpel, enhancing carpel growth and therefore encouraging out-crossing. On the other hand, JMJ13 activates genes of the jasmonic acid regulatory network alongside the auxin responsive SAUR26, to inhibit carpel growth, enhance stamen growth, and overall promote self-pollination. Our evidence provides novel mechanisms of self-fertility regulation in *A. thaliana* demonstrating how chromatin modifying enzymes govern the equilibrium between flower self-pollination and out-crossing.

## Introduction

As a predominantly self-fertilizing plant, the growth and development of the male and female organs in the Arabidopsis flower need to be coordinated. How this is achieved is not yet fully understood (Wellmer et al., 2013). Since chromatin regulation is involved in almost every developmental process of a plant's life cycle, we investigated how it contributes toward the correct timing of floral organ development to enable self-fertility. A particular histone modification that is highly dynamic throughout Arabidopsis development is the trimethylation of the lysine 27 residue of histone 3 (H3K27me3) which induces transcriptional silencing (Francis et al., 2004; Entrevan et al., 2016; Frerichs et al., 2019). H3K27me3 is deposited at thousands of Arabidopsis genes by the polycomb repressive complex 2 (PRC2) (Zhang et al., 2007; Lafos et al., 2011) and represses floral development genes in the seedling (Wang et al., 2016). Accordingly, at some developmental stage these floral development genes must be reactivated by removal of H3K27me3 and addition of active chromatin marks such as H3K4me3 or H3K36me3 (Pfluger and Wagner, 2007).

Three genes have been demonstrated to encode targeted H3K27me3 specific demethylases which may reactivate these floral development genes; ELF6, REF6, and JMJ13 (Lu et al., 2011; Crevillén et al., 2014; Yan et al., 2018). Recent findings suggest that each demethylase is recruited to a large number of target genes, some of which are targeted by more than one demethylase (Yan et al., 2018; Antunez-Sanchez et al., 2020). Recruitment to these genes is achieved by a combination of direct DNA binding via a Zinc finger domain and interaction with other transcription factors (Yu et al., 2008; Li et al., 2016; Yan et al., 2018). Certain physiological functions have already been assigned to the three demethylases, such as regulation of flowering time (Zheng et al., 2019), control of leaf cell elongation (Yu et al., 2008) and resetting the epigenome across generations (Crevillén et al., 2014; Antunez-Sanchez et al., 2020; Borg et al., 2020). Functions such as controlling leaf cell elongation require specific targeting of the demethylases to a subset of their global target genes. In the context of leaf cells, this is achieved by the interaction of ELF6 and REF6 with the BZR2 transcription factor which recruits the demethylases to specific target genes (Yu et al., 2008). Furthermore, in floral buds REF6 has been demonstrated to interact with a number of developmentally important MADS-box transcription factors (Yan et al., 2018).

The role of the demethylases in epigenetic reactivation of floral development genes and thus control of floral development is poorly understood. Though changes to floral morphology have been observed in *elf6 jmj13 ref6* triple mutants (Yan et al., 2018), the function of each individual demethylase in floral development and self-pollination control is unknown. In this study we reveal that two of the histone demethylases, ELF6 and JMJ13, antagonistically regulate self-pollination by modulating the growth of stamen and carpel, linking ELF6/JMJ13-dependent chromatin regulation to floral development and self-fertility. We further investigate the transcriptome and epigenome changes caused by loss of these demethylases to predict the target genes which may be responsible for these novel developmental functions.

## Materials and Methods

### Plant Material

*A. thaliana* Columbia-0 ecotype (Col-0) was used in this study as wild-type material. All knock-out mutants were T-DNA insertions of Col-0: *jmj13* (GABI_113B06), *elf6-3* (SALK_074694C), *ref6-1* (SALK_001018C). Double and triple mutants were generated by crossing and genotyping (kindly provided by Prof C Dean, John Innes Centre, UK). pJMJ13::JMJ13-GFP in *jmj13* genetic background and p35S::ELF6-GFP in *elf6* genetic background (Kindly provided by Dr. H Yang and Prof C Dean, John Innes Centre, UK) were used for phenotypic complementation.

### Fertility Assessment

The number of failed siliques was counted on the primary inflorescence after approximately 6 weeks of growth such that at least 10 siliques had matured on each primary inflorescence sampled.

### Floral Organ Phenotype Measurements

Col-0, *elf6, jmj13, ref6*, all double and triple mutants were grown for 4–5 weeks until flowering. Stage 14 buds, as defined by Smyth et al. (1990), corresponded to the 2 youngest open buds of the inflorescence and were used as samples for floral organ height quantification. Buds were imaged using either the Leica MZ165 or the Olympus SZ61. ImageJ (Rueden et al., 2017) was used to perform measurements of stamens and carpels.

### Pollen Viability Assay

Pollen from stage 14 flowers the first four flowers were stained with 5 ug/ml of fluorescein diacetate to examine pollen viability (*n* = 5 flowers per genotype, *n* > 150 pollen grains per genotype).

### Transcriptome Sequencing

RNA was extracted from stage ~9–13 buds (Smyth et al., 1990) by phenol-chloroform extraction (Box et al., 2011). Single-end deep sequencing of two replicates from each genotype was performed after mRNA enrichment (BGI technology). Sequences (>30 million from each sample) were aligned to the TAIR10 genome using bowtie2 (Langmead and Salzberg, 2012) and differentially expression analysis performed using NOISeq (Tarazona et al., 2011). Differentially expressed genes are defined by a 2-fold change in expression and a NOISeq probability score ≥ 0.8.

### Ploidy Analysis

Ploidy analysis method was adapted from Yang et al. (2019). Sixteen carpels for each genotype were dissected from stage 14 flowers at inflorescence positions 1 and 2. The dissected carpels were immediately placed in ~400 ul of nuclei isolation buffer (0.01 M MgSO4, 0.05 M KCl, 1.2 mg/ml HEPES buffer, 10 mM DTT, 2.5% Triton-X100 in water). Carpel tissue was then diced in the buffer to release the nuclei and was filtered through a double layer of Mira-cloth (pore size 22–25 μm). DAPI was added to a final concentration of 2 μg/ml and incubated at room temperature for 15 min. This solution (400 μl) was then run through the BD LSR Fortessa™ flow cytometer and DAPI was excited with a 405 and 640 nm laser and light collected at 450 nm+/−25 and 780 nm+/−30, respectively.

### Cell Elongation Analysis

Flowers from Col-0 (*n* = 4), *elf6* (*n* = 3), and *jmj13* (*n* = 2) were sampled at stage 14 from the first flower position on the inflorescence. Each flower was dissected to leave just the intact gynoecium attached to the stem. The gynoecium was cleared in 80% isopropanol for between 110 and 130 min and then stained in 20 μg/ml propidium iodide (PI) solution for 20 min. The PI stained gynoeciums were imaged with a Leica SP5 upright confocal microscope with an excitation wavelength of 514nm and an emission capture range of 585–602 nm. Cross-sections of the upper and lower carpel were imaged. The cells of the outer epidermal cell files of the carpel were measured along the long axis of the carpel in ImageJ (Rueden et al., 2017), measuring at least 95 cells per genotype.

### General Data Analysis

The R statistical programming language (R Core Team, 2019) was used for all data analysis and graph generation excluding the flow cytometry data which was analyzed in FCSalyzer (https://sourceforge.net/projects/fcsalyzer/). The following R packages were utilized in the analysis and graphing of data: tidyverse (Wickham et al., 2019), eulerr (Larsson, 2020), BioMaRt (Durinck et al., 2009).

### Gene Accession Numbers

*A. thaliana* gene locus identification codes for genes mentioned in this study (for the full list of predicted target genes see Supplementary Table 1) are as following: ELF6 (AT5G04240); REF6 (AT3G48430); JMJ13 (AT5G46910); BZR1 (AT1G75080); BZR2 (AT1G19350); INO (AT1G23420); MYB24 (AT5G40350); SMR8 (AT1G10690); EXPA1 (AT1G69530); EXPA3 (AT2G37640); EXPB3 (AT4G28250); SAUR26 (AT3G03850); AGP14 (AT5G56540); AGP22 (AT5G53250); AGP7 (AT5G65390).

### Data Availability

All RNA sequencing datasets generated in this study can be accessed through NCBI Gene Expression Omnibus (GEO; https://www.ncbi.nlm.nih.gov/gds) under accession number GSE164739.

## Results

### ELF6 and JMJ13 Antagonistically Regulate Arabidopsis Self-Fertility via Floral Organ Growth

**Figure 1:**
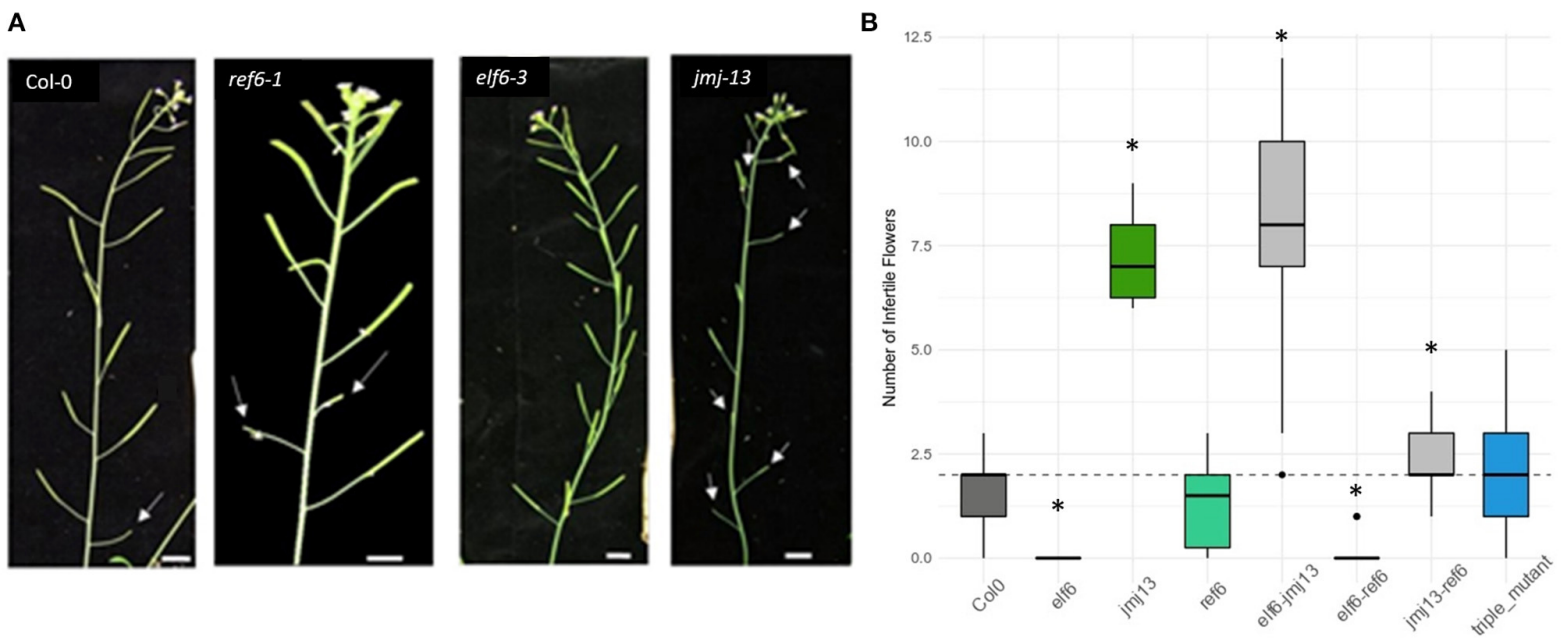
Fertility phenotypes of H3K27me3 demethylase knock-out mutants. **(A)** Varying degrees of self-fertility in the demethylase knock-out mutants. The white arrow heads point to aborted siliques caused by failed self-pollination, as seen in the first flower of Col-0 and to the eighth flower of *jmj13*. Scale bars represent 1 cm. **(B)** Quantification of self-fertility. Numbers of aborted siliques are plotted with asterisks indicating which data point is statistically significant as compared to that of Col-0 (*p* < 0.05, *t*-test). The dashed horizontal line represents the median *y*-axis value of Col-0.

**Figure 2:**
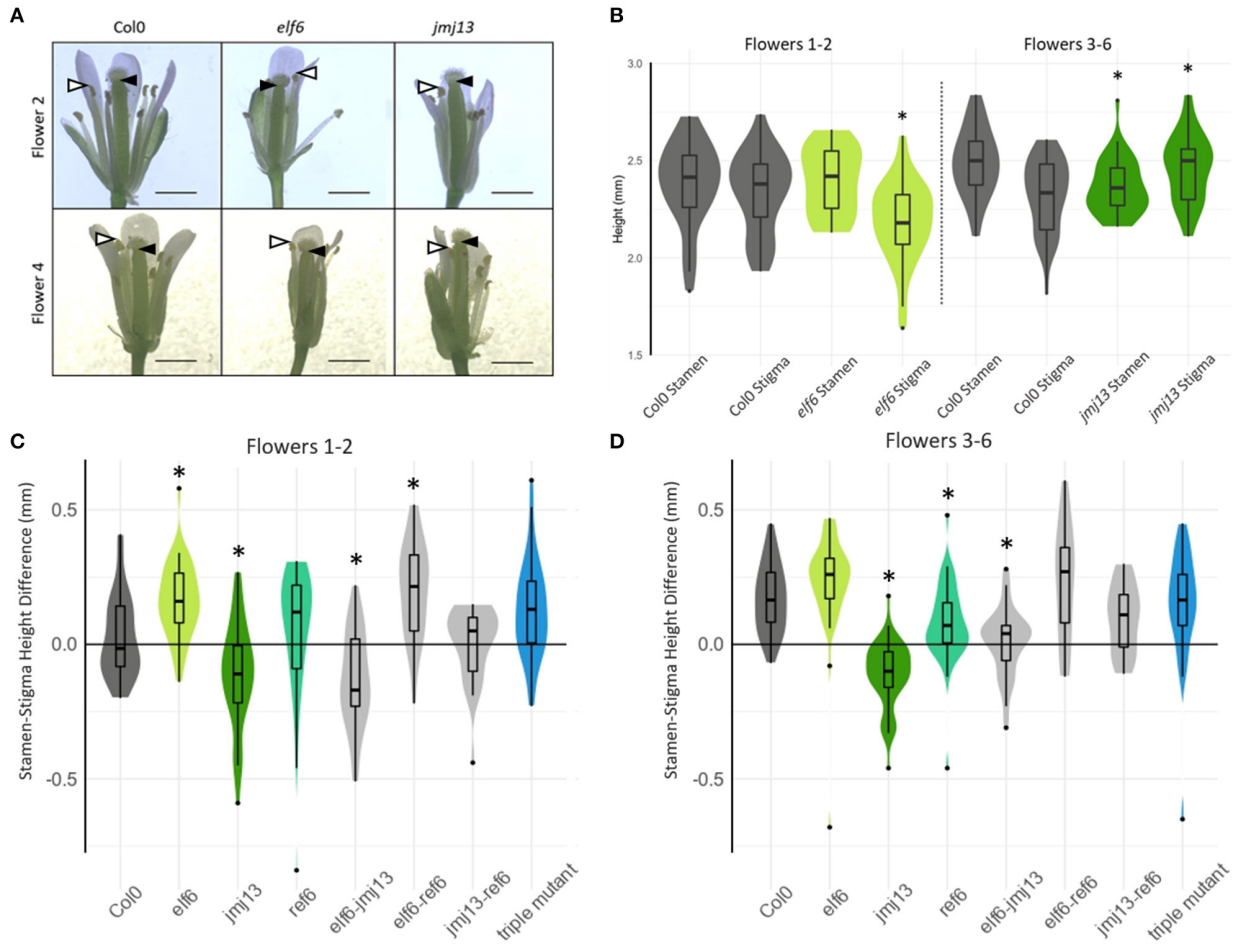
Quantification of floral organ lengths in H3K27me3 knock-out mutants. **(A)** Representative images of stage 14 flowers of the indicated genotypes from flower positions 2 and 4 (scale bars represent 1 mm). Black arrow heads indicate the lowest point of the stigma closest to the receptacle, whilst white arrow heads indicate highest point of the tallest stamen, the difference of which correlates with self-fertility. **(B)** The absolute heights of stamen and stigma from Col-0, *elf6*, and *jmj13* flowers. Measurements were taken from receptacle to the top of the highest stamen and to the bottom of the stigma. *n* >= 20 for each genotype in each flower position. An asterisk indicates statistical significance compared to the respective Col-0 data of the same floral organ and flower position group (*p* <= 0.05, *t*-test). **(C)** The lengths from receptacle to the tip of the tallest stamen, and receptacle to the lowest point of the stigma were measured. The difference between these two lengths is plotted for every flower measured at flower positions 1–2 and distributed by genotype. Groups marked with an asterisk are statistically significantly different from the Col-0 stamen/stigma height differences (*p* < 0.05, *t*-test). The violin graph represents the density of data point distribution displayed symmetrically along the y axis. *n* >= 20 for each genotype. **(D)** Stamen-stigma height differences were measured and plotted as in **(C)** but for flower positions 3–6. *n* >= 20 for each genotype.

Fertility in the context of Arabidopsis refers to the ability of a flower to generate a full-length silique with viable seeds. Arabidopsis thaliana is predominantly self-fertilizing, in that the female gynoecium of a typical flower is fertilized by pollen from the same flower. However, the first two flowers to mature often fail to self-fertilize resulting in stunted siliques (Figure 1A). The infertility in these first two flowers is caused by a failure to self-pollinate, as the stigma extends beyond the reach of the mature stamens preventing pollen transfer from anther to stigma (Figure 2A). By manually fertilizing flowers with their own pollen, we restored fertility confirming that the absence of self-fertility is not caused by gamete viability but by failure to transfer pollen (Supplementary Figure 1).

To reveal the role of the H3K27me3 demethylases in regulating self-pollination, the number of infertile siliques was quantified in *elf6, ref6*, and *jmj13* T-DNA mutants (Figure 1) (see methods for specific alleles). In *elf6* and *jmj13*, significant and opposite changes to the degree of self-fertilization were observed. *elf6* displayed increased self-fertility whereby all flowers, even the first two flowers, were consistently self-fertile. Conversely, an infertility phenotype was observed in *jmj13* whereby aborted siliques were observed all the way to the eighth flower of the primary inflorescence. ref6 did not show a significant change in fertility. The relationship between the demethylases was also probed by quantifying fertility in double and triple mutant combinations. Double mutants displayed non-obvious floral phenotypes; *elf6 jmj13* adopted a *jmj13*-like reduced fertility, *elf6 ref6* was super-fertile while *jmj13 ref6* showed an intermediate phenotype between *jmj13* and *ref6*. Finally, the triple mutant showed no significant change from the wild-type (Figure 1B, Supplementary Figure 2).

To confirm that the fertility phenotypes are attributed to loss of the demethylases rather than artifacts of random T-DNA insertions, stable complementation lines expressing p35S::ELF6-GFP and pJMJ13::JMJ13-GFP in *elf6* and *jmj13* mutant backgrounds, respectively, were characterized. Both transgenic lines restored fertility to wild-type levels (Supplementary Figure 3). We next questioned whether the infertility phenotype of *jmj13* may be caused by a reduction in pollen viability. Pollen from Col-0*, elf6* and *jmj13* was stained with fluorescein diaecetate in which only viable pollen displays fluorescein fluorescence. No significant change in pollen viability was found between Col-0 and *jmj13* or *elf6* (Supplementary Figure 4). To verify that male and female reproductive organs were still individually functional in the absence of JMJ13, manual self-pollination was performed. Fertility of the first two flowers was restored in Col-0 and *jmj13* when they were manually self-pollinated (Supplementary Figure 1, *n* ≥ 36).

We hypothesized that these changes in fertility upon loss of H3K27me3 demethylases were due to changes in carpel and stamen growth altering the probability of self-pollination. To assess this hypothesis, the lengths of carpel and stamen were measured from flowers at developmental stage 14 when self-pollination typically occurs in Col-0 (Smyth et al., 1990) (Figure 2A). Two lengths were measured: from the receptacle (base of the flower) to the top of the tallest stamen and from the receptacle to the bottom of the stigma (Figure 2B). The difference between these two lengths was calculated such that positive values indicate that the stamens were taller than the stigma and self-pollination was likely to occur, whereas negative values indicate the stamens were shorter than the stigma and the flower was likely to fail in self-pollination. As expected, Col-0 displayed a negative median floral organ height difference in the first two flowers and positive median difference in flowers 3–6 (Figure 2A) as is consistent with the typical fertility of flowers in those positions (Figure 1B). The stamen-stigma height differences in the single mutants were also consistent with their fertility phenotypes. The “super-fertile” *elf6* displayed a positive median stamen/stigma height difference in all flower positions and was significantly greater than Col-0 in flowers 1–2 (*p* < 0.05, *t*-test); the semi sterile *jmj13* median stamen/stigma difference was negative and significantly less than Col-0 in all flower positions measured (*p* < 0.05, *t*-test). *ref6* median stamen-stigma difference showed no statistically significant difference from Col-0 in flowers 1–2. Specifically, the absolute heights of stamen and stigma show that *elf6* had significantly shorter carpels than Col-0 in flowers 1–2 whilst *jmj13* had significantly longer carpels and shorter stamens than Col-0 in flowers 3–6 (Figures 2B,C). The data demonstrates that JMJ13 and ELF6 play crucial antagonistic roles in regulating self-fertility, by regulating the growth of specific floral organs.

### Transcriptome Wide Changes Occur in *elf6* and *jmj13* Mutants

**Figure 3:**
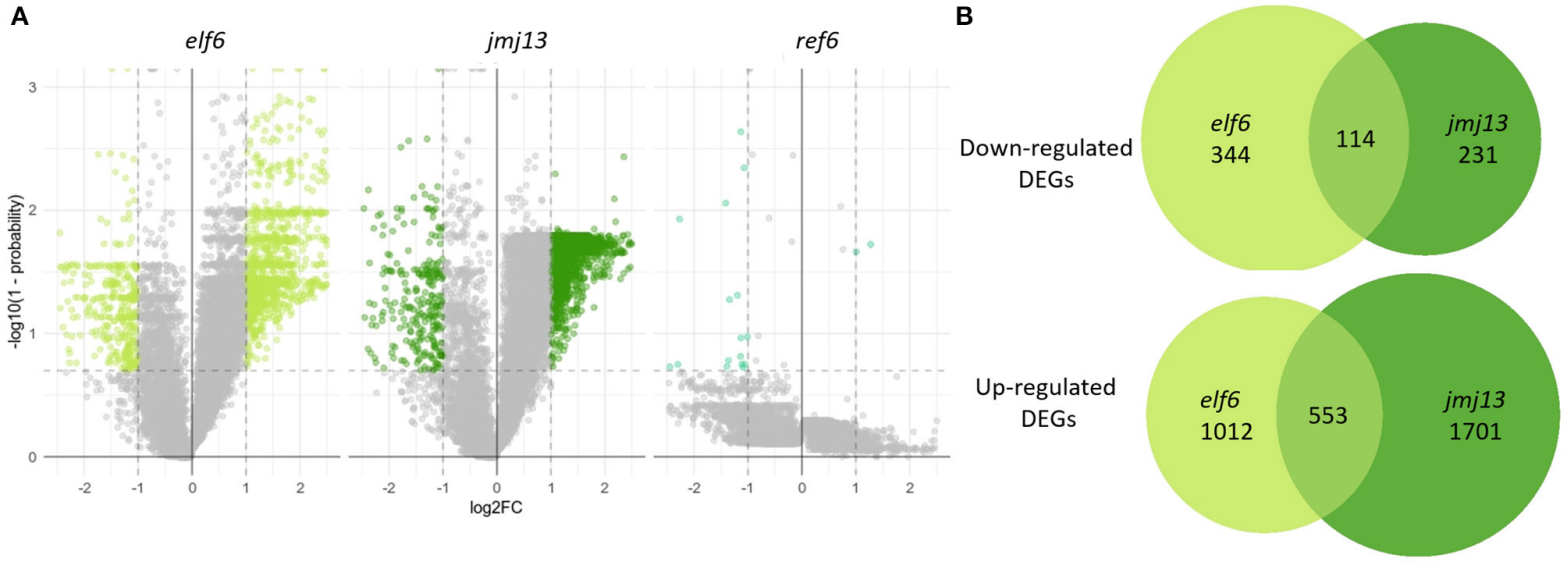
Transcriptome changes in the bud in *elf6, ref6* and *jmj13* knock-out mutants compared to Col-0. **(A)** Transcriptomes of *elf6, jmj13*, and *ref6* buds compared after differential expression analysis against the transcriptome of Col-0 buds. The significance thresholds drawn in dashed lines represent a 2-fold change in expression and a probability score > 0.8. Genes over these thresholds are classed as differentially expressed and colored in lime green, forest green and cyan dependent on the genotype. **(B)** DEGs extracted from the *elf6* and *jmj13* transcriptomes. They are split into up and down regulated DEGs and represented as individual venn groups, with the shared group representing genes that are either differentially up or down regulated in both *elf6* and *jmj13* buds compared to Col-0.

To understand the role of H3K27me3 demethylation in regulating floral organ growth and self-pollination, transcriptome datasets were generated from stage ~9–13 buds of Col-0, *elf6, jmj13*, and *ref6* inflorescences. Large transcriptome changes were observed in *elf6* and *jmj13*; defined by > 2-fold expression change and > 0.8 NoiSeq probability score, there were 2,023 and 2,599 differentially expressed genes (DEGs), respectively, when compared to Col-0 (Figure 3). The *ref6* knock-out buds on the other hand displayed only minor transcriptome changes as only 103 DEGs were observed, consistent with the subtle fertility phenotype of *ref6*. There is proteomic evidence to show that REF6 binds to a number of important MADS-box transcription factors controlling floral development (Yan et al., 2018) and so it is unexpected that *ref6* displays the weakest floral development phenotype and transcriptome effects. One possible explanation may be that the T-DNA insertion in the *ref6-1* mutant (SALK_001018C) does not completely inhibit REF6 function. This may be because in this *ref6-1* mutant the catalytic domain remains intact and only the zinc-finger domain is disrupted by the inserted T-DNA. Previous studies (Yan et al., 2018) have shown that REF6 still targets several thousand genes without its zinc-finger domain, merely losing specificity, and so *ref6-1* likely also retains targeting and catalytic ability. Read alignment to the *ref6* gene model confirmed that the catalytic domain is still expressed in the *ref6* T-DNA insertion mutant (Supplementary Figure 5). This is in contrast to the *elf6* and *jmj13* mutants in which the catalytic domain is completely disrupted by T-DNA insertion.

The *elf6* and *jmj13* transcriptome datasets were further analyzed to gain a mechanistic understanding of self-fertility regulation by the H3K27me3 demethylases. DEGs from both the *elf6* and *jmj13* datasets contained mostly up-regulated genes. This demonstrates that the majority of DEGs are not direct floral targets of the demethylases as a floral target gene would remain epigenetically silenced without the respective H3K27me3 demethylase due to ectopic accumulation of H3K27me3. Significant, but minority, overlap was found between the *elf6* and *jmj13* DEGs (3230 unique DEGs, 696 shared DEGs, *p* < 0.0001).

A data screening approach was taken to predict ELF6 and JMJ13 target genes responsible for the floral organ growth phenotypes. We first assumed that without ELF6 or JMJ13, their respective target genes would become ectopically enriched in H3K27me3. Using a H3K27me3 ChIP-seq dataset from Col-0 and demethylase triple mutant buds (Yan et al., 2018) we selected all genes which showed significant H3K27me3 enrichment in the triple mutant buds (3,216 genes). The phenotypic target genes would also be expected to be transcriptionally silenced in the *elf6* or *jmj13* mutant due to H3K27me3 accumulation. From our RNAseq data, 61 and 42 genes from the 3,216 gene subset were significantly down-regulated in *elf6* and *jmj13*, respectively. This gene list comprises our predicted ELF6 and JMJ13 target genes. To further reduce this list to the most likely candidates it was assumed that because *elf6* and *jmj13* show opposing fertility and carpel growth phenotypes that the genes responsible should be differentially expressed between the *elf6* and *jmj13* mutants. 41 of the 103 predicted ELF6/JMJ13 target genes showed a 2-fold change in expression between *elf6* and *jmj13* (Supplementary Table 1). From this subset, genes of unknown function were discarded and the remaining 18 ELF6 target genes (Table 1) and 11 JMJ13 target genes (Table 2) were determined to be the most likely ELF6/JMJ13 target genes causing the observed phenotypes.

**Table 1:** Predicted ELF6 phenotypic target genes as determined by screening of transcriptome and epigenome data.

**ELF6 target gene name**	**Target gene function**	**Log2(*elf6*/Col-0)**	**Log2(*jmj13*/Col-0)**
EXPA1	Promotes cell elongation by cell wall loosening (Cosgrove 2015)	−1.26	0.47
EXPA3	Promotes cell elongation by cell wall loosening (Cosgrove 2015)	−1.51	−0.49
EXPB3	Promotes cell elongation by cell wall loosening (Cosgrove 2015)	−1.01	0
ADP1/ABS3	Promotes cell elongation, branching, sensecence and fertility in auxin dependent manner (Li et al., 2014; Wang et al., 2015)	−1.1	−0.16
PRR9	Regulation of the circadian clock and flowering time (McClung and Gutiérrez, 2010)	−1.22	1.02
ZFP2	Negatively regulates floral abscission and stamen length (Cai and Lashbrook, 2008)	−3.19	−1.2
CBF1	Transcriptional activator of cold tolerance and ABA response genes (Li et al., 2017)	−2.76	0.38
CYP81F4	Secondary metabolite synthesis	−1.66	0.16
MARD1	Regulator of ABA mediated seed dormancy and interactor of SnRK1 autophagy activator (He and Gan, 2004; Nietzsche et al., 2014)	−1.39	0.63
LEA2	Promotes root growth and drought tolerance (Magwanga et al., 2018)	−1.8	−0.52
EPFL2	Stomata guard cell differentiation	−1.07	0.67
FLZ7	Adaptor for SnRK1 autophagy regulation (Jamsheer et al., 2018)	−2.12	−1.01
ATL98	Ubiquitin mediated protein degradation	−2.3	0.09
MBOAT	Membrane bound O-acyl transferase	−1.05	0.19
PLC6	Phosphoinositol-DAG signaling	−1.1	0.26
AT2G29660	Zinc-finger transcription factor	−1.07	0.12
CUAOy2	Amine metabolism	−1.3	0.47
DTX49	Xenobiotic efflux	−1.5	−0.24

**Table 2:** Predicted JMJ13 phenotypic target genes as determined by screening of transcriptome and epigenome data.

**JMJ13 target gene name**	**Target gene function**	**Log2(*elf6*/Col-0)**	**Log2(*jmj13*/Col-0)**
JAZ7	Regulator of jasmonic acid signaling, flower development, flowering time, drought tolerance, pathogen defense (Browse and Wallis, 2019)	1.43	−1.77
SAUR26	Auxin responsive positive regulator of cell elongation, enriched expressed in stamens (Spartz et al., 2014)	−0.19	−2.13
AGP22	Membrane proteoglycan involved in cell-cell signaling and wound response (Guan and Nothnagel, 2004)	−1.1	−2.29
AGP14	Membrane proteoglycan involved in cell-cell signaling and wound response (Guan and Nothnagel, 2004)	0.25	−1.17
AGP7	Membrane proteoglycan involved in cell-cell signaling and wound response (Guan and Nothnagel, 2004)	0.64	−1.2
LEC	Lectin up-regulated by chitin, mechanical damage, jasmonic acid, ethylene	2.24	−2.24
SAQR	Regulator of flowering time and starch allocation (Jones et al., 2016)	−0.1	−3
XTH24	Cell wall expansion by xyloglucan cleavage and re-ligation (Lee et al., 2018)	0.46	−1.37
PUP18	Purine and possibly cytokinin transporter, induced by the AP3 and PI homeotic transcription factors (Mara and Irish, 2008)	1.8	−1.94
ATL89	Ubiquitin mediated protein degradation	−0.14	−1.94
UPS4	Ureide permease	−0.31	−2.45

### ELF6 Likely Relieves Expansin Genes of H3K27me3 Suppression to Induce Cell Elongation

Of the 18 predicted phenotypic target genes of ELF6, three were identified as expansins (EXPB3, EXPA3, and EXPA1) (Supplementary Figure 8). The expansins are cell wall remodeling enzymes which disrupt non-covalent bonding between cellulose microfibrils to relax the cell wall allowing turgor-pressure to induce cell-elongation (Cosgrove, 2015). Expansins induce cell elongation responses in numerous developmental contexts (Marowa et al., 2016), but have yet to be implicated in carpel growth. It is likely that the loss of ELF6 has caused multiple expansin genes to remain epigenetically silenced by H3K27me3 and thus unable to induce cell elongation in the carpel, retarding carpel growth. We next asked how ELF6 might be recruited to the expansin genes. The expansins are shown to be regulated by the brassinosteroid response transcription factor BZR2 which has also been demonstrated to directly bind ELF6 and REF6 (Yu et al., 2008, 2011). It is likely that in the carpel BZR2 recruits ELF6 to multiple expansin genes. Supporting this theory, we found that our 60 predicted ELF6 target genes are significantly enriched in ChIP-seq validated target genes of BZR2 and its close homolog BZR1 whereas the predicted JMJ13 target genes showed no such enrichment (ELF6-BZR2, x2.26 enrichment, *p* = 0.049; ELF6-BZR1, x1.78 enrichment, *p* = 0.026, hypergeometric test, Supplementary Figure 6).

**Figure 4:**
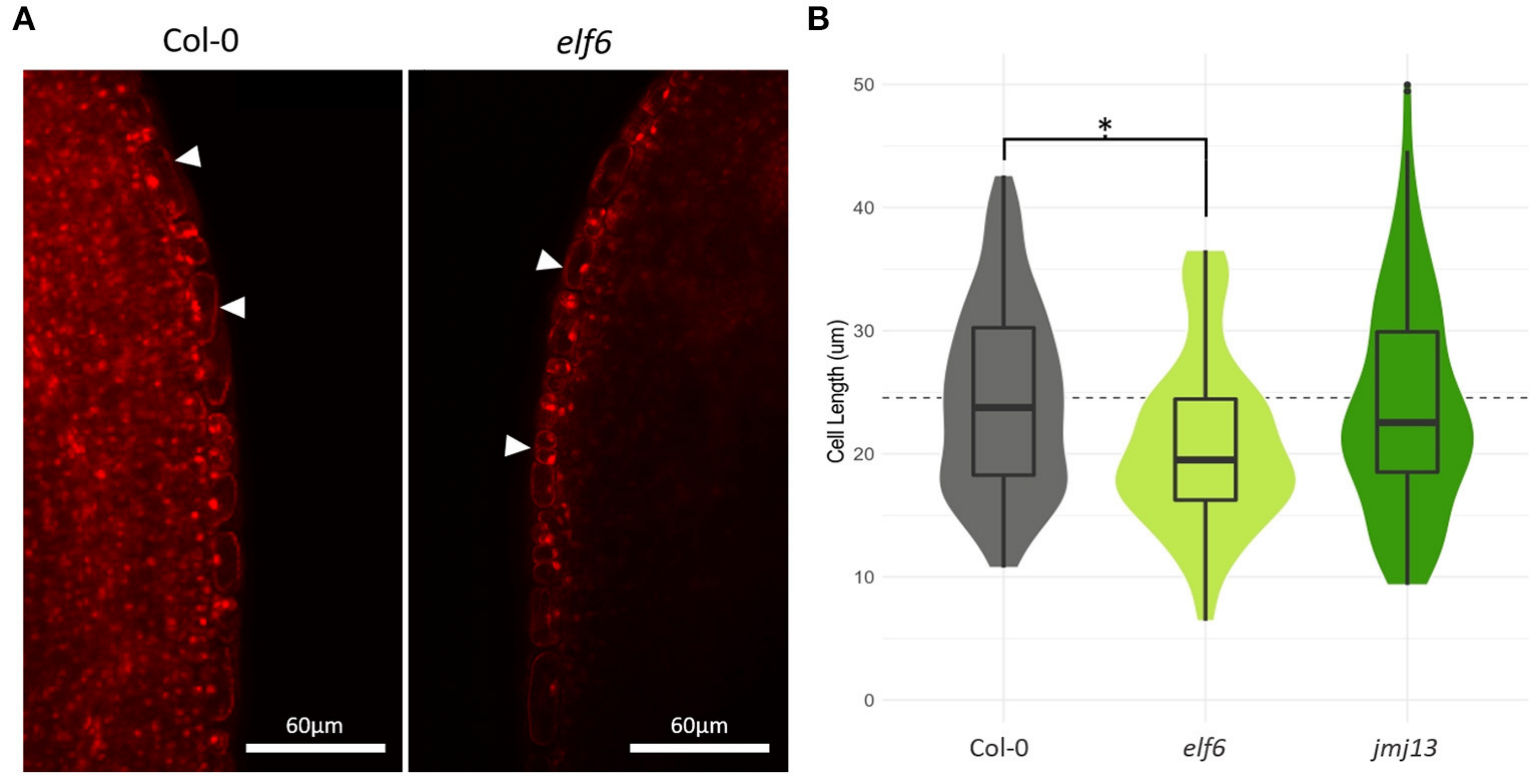
Effects of *elf6* knock-out on carpel epidermal cell length. **(A)** Confocal microscopy of stage 14 carpel mid sections using propidium iodide staining to highlight individual cells. The image on the left is taken from a Col-0 carpel, whilst the right hand image is from *elf6*. White arrow heads indicate the epidermal cell layer. Scale bar depicts 60 μm. **(B)** Measurements of cell length in carpel epidermis. A significant 15% reduction in mean epidermal cell length was observed in *elf6* carpels compared to Col-0 carpels (*n* >= 3 carpels per genotype, *n* >= 95 cells measured per genotype, *p* <= 0.05, *t*-test). The dashed line represents the mean carpel epidermal cell length of Col-0 carpels. The asterisk denotes a significant change in cell length between the indicated genotypes (*p* <= 0.05, *t*-test).

If expansin epigenetic silencing in *elf6* is the primary cause of stunted carpel growth, we should observe a reduction in cell size in the *elf6* carpel. Cell elongation is a key growth mechanism for the maturing silique (Ripoll et al., 2019). However, it is unclear whether differential cell elongation at the self-pollination stage can affect the chances of successful pollen deposition onto the stigma. Using confocal microscopy, we measured cell length in the carpel epidermis of stage 14, position 1 flowers from Col-0, *elf6* and *jmj13* plants (Figure 4). A significant 15% decrease in mean cell length was observed in *elf6* carpels compared to Col-0 carpels (n carpels ≥ 3, n cells ≥ 95, *p* <= 0.05, *t*-test), from a mean cell length of 24.5 μm in Col-0 (SD = 7.6) to 20.8 μm in *elf6* (SD = 6.9). This reduction in cell length could explain the ~8% reduction in total carpel length of position 1 flowers (Col-0 mean length = 2.51 mm, SD = 0.15; *elf6* mean length = 2.32 mm, SD = 0.10) (Figure 2B), therefore, supporting a role for the expansins in promoting carpel growth to prevent self-pollination.

Our bioinformatic pipeline also proposed the cyclin dependent kinase inhibitor SMR8, a likely regulator of endoreduplication, to be targeted by ELF6 (Van Leene et al., 2010) (log2FC(*elf6*/*jmj13*) = −0.9, so not featured in Table 1). We hypothesized that endoreduplication, mediated by SMR8, may be a synergistic growth mechanism in the wildtype carpel and that loss of ELF6 could silence SMR8 thus inhibiting endoreduplication and carpel growth. To verify this hypothesis, the ploidy state of stage 14 gynoecium nuclei was assessed using DAPI staining and flow cytometry. No significant endoreduplication could be detected in either Col-0, *elf6*, or *jmj13* gynoecia, concluding that endoreduplication is unlikely to be employed in carpel growth up to floral developmental stage 14 and is not affected by ELF6 or JMJ13 in the carpel (Supplementary Figure 7).

### JAZ7, SAUR26 and Multiple Arabinogalactan Proteins Are Silenced in jmj13 Buds

Of particular interest in the set of predicted JMJ13 phenotypic target genes is the jasmonic acid response transcription factor JAZ7 (Supplementary Figure 8). Jasmonic acid is a key hormonal regulator of floral organ development and acts by binding JAZ repressor proteins to induce their degradation (Huang et al., 2017). JAZ proteins bind and inhibit other developmental transcription factors (Song et al., 2011) such as YABBY and MYB transcription factors to bring about controlled floral development (Meister et al., 2005; Reeves et al., 2012; Boter et al., 2015; Qi et al., 2015; Gross et al., 2018). Two members of the YABBY family in particular, CRC and INO, are involved in gynoecia development and both display stunted carpel growth in knock-out mutants (Meister et al., 2005). INO was found to be significantly up-regulated in the *jmj13* inflorescence (1.4 log2FC, 0.98 probability score) which may be considered phenotypically consistent with the enlarged gynoecia observed in *jmj13* mutants (Figure 2B). However, it is not clear whether this dysregulation of INO expression is related to the ectopic epigenetic silencing of JAZ7.

The other common JAZ targets, the MYB transcription factors, are also heavily involved in floral development. MYB21 and MYB24 in particular are known to promote stamen elongation and *myb21-myb24* knock-out mutants display a reduced stamen/gynoecium length ratio and significantly decreased fertility in a very similar manner to the *jmj13* knock-out phenotype (Reeves et al., 2012; Qi et al., 2015). MYB24 was found to be significantly down-regulated in the *jmj13* knock-out inflorescence (−1.6 log2FC, 0.99 probability score), providing a phenotypically consistent hypothesis to explain the reduced stamen growth and subsequent infertility of *jmj13* knock-out flowers. However, there were no significant changes to the H3K27me3 profile across the MYB24 gene, indicating that MYB24 must be indirectly repressed downstream of a JMJ13 target. Several JAZ proteins (JAZ1, JAZ8, JAZ11) have been shown to bind the MYB family transcription factor MYB24 (Song et al., 2011) and so it may be possibly be a target of JAZ7 too. Though there is lacking evidence to tie together the observed ectopic H3K27me3 silencing of JAZ7 and the gene expression changes of MYB24 and INO, it is clear that JMJ13 is playing an important role within this regulatory network.

In addition to the jasmonic acid regulator JAZ7, the auxin response gene SAUR26 is also epigenetically silenced in *jmj13* (Supplementary Figure 8). Numerous SAUR genes have been demonstrated to induce cell elongation via cell wall acidification (Spartz et al., 2014) and the SAUR63 subfamily has been specifically demonstrated to induce stamen elongation (Chae et al., 2012). Though the role of SAUR26 in stamen elongation has not been studied, SAUR26 does show highly enriched expression in the stamens and so is likely to serve the same function as the SAUR63 subfamily (Klepikova et al., 2016).

A final major class of potential JMJ13 target genes is the arabinogalactan proteins (AGPs); highly glycosylated proteins of the outer plasma membrane implicated in developmental signaling (Seifert and Roberts, 2007). Three AGPs were predicted to be phenotypic targets of JMJ13; AGP7, AGP14, and AGP22. However, the biochemical function and physiological role of specific AGPs is poorly understood and difficult to relate to floral organ growth. Interestingly, perturbation of AGPs elicits a wound-like response (Guan and Nothnagel, 2004) hinting at a possible connection between JMJ13, AGPs and the jasmonic acid regulatory network which also regulates wound response.

## Discussion

Stamen/carpel growth coordination is differentially regulated according to the position of flowers along the length of the inflorescence, such that a small portion of flowers refrain from self-fertilization (Plackett et al., 2017). Despite failing to self-pollinate, the stamen and gynoecium of these first flowers are still fully functional and able to produce viable siliques if pollen is artificially transferred from anther to stigma (Supplementary Figure 1). It is likely that *A. thaliana* in the wild receives some form of evolutionary advantage by refraining from self-pollination in the first flowers. In the wild, pollinators are likely to cause pollen transfer and thus enable out-crossing in these first two flowers providing evolutionary benefits in the form of genetic diversity. However, self-pollination is a far safer reproduction strategy as it is less dependent on external pollinators and resources are not invested by the plant to attract pollinators as with other species. Therefore, by self-pollinating all but the first two flowers, *A. thaliana* likely optimizes the benefits of out-crossing and self-fertilization (Stebbins, 1974; Wright et al., 2013). Our evidence demonstrates that the histone demethylases ELF6 and JMJ13 antagonistically regulate this evolutionary equilibrium between self-fertilization and out-crossing. We find that ELF6 reactivates floral development genes to promote carpel growth and outcrossing, whilst JMJ13 reactivates a different set of floral development genes to inhibit stamen growth and promote carpel growth to stimulate self-pollination.

Transcriptome and epigenome data (Yan et al., 2018) from flowers of demethylase mutants has been used to specifically identify the likely target genes causing these floral organ growth effects. Our findings suggest that ELF6 epigenetically activates multiple expansin genes, via BZR2 recruitment, inducing carpel cell elongation. The identification of JAZ7 as a JMJ13 target gene implicates chromatin regulation as a mechanism mediating the jasmonic acid gene regulatory network. The current literature is unclear on what the downstream effects of JAZ7 epigenetic silencing might be, largely due to there being multiple possibly redundant JAZ genes hindering studies on single gene knock-out mutants (Wager and Browse, 2012). Our observation of MYB24 down-regulation provides a phenotypically consistent explanation as to why *jmj13* displays stunted stamens and decrease fertility, but MYB24 does not show H3K27me3 enrichment in the demethylase triple mutant implying that it is not a direct demethylase target gene. Similarly with INO; although INO and its binding partner CRC are known to play a role in promoting carpel growth (Gross et al., 2018), INO is clearly not a direct target of JMJ13 as it is up-regulated in the *jmj13* mutant. As JAZ7, MYB24, and INO are part of the same regulatory network, it is possible that an uncharacterised intermediate connects JAZ7 function to transcriptional regulation of MYB24 and INO. Moreover, the complex feedback of the floral development regulatory network suggests that ectopic epigenetic silencing of a single gene such as JAZ7 may induce unexpected changes to gene expression throughout the network (Reeves et al., 2012). An alternative or synergistic hypothesis linking JMJ13 to stamen growth is the epigenetic activation of SAUR26, a gene likely to induce stamen elongation via cell wall acidification (Chae et al., 2012).

We have demonstrated that the histone demethylases ELF6 and JMJ13 epigenetically regulate distinct sets of floral development genes to regulate floral morphology. Our evidence supports mechanisms whereby ELF6 promotes carpel elongation via epigenetic activation of expansin genes, whilst JMJ13 represses carpel growth via jasmonic acid signaling and promotes stamen growth via epigenetic reactivation of SAUR26. These conclusions establish histone demethylation as a key mechanism in regulating the chromatin state of floral development genes and hence controlling floral morphology and the equilibrium between self-pollination and out-crossing.

## Data Availability Statement

The datasets presented in this study can be found in online repositories. The names of the repository/repositories and accession number(s) can be found at: NCBI Gene Expression Omnibus, accession no: GSE164739”.

## Author Contributions

CK, BM, and JS contributed to the conception and design of the study. CK, BM, ET, SJ, and JS performed the experiments and analyzed the data. CK performed all bioinformatics analysis and drafted the manuscript. CK, BM, and JS revised and edited the manuscript. All authors contributed to the article and approved the submitted version.

## Conflict of Interest

The authors declare that the research was conducted in the absence of any commercial or financial relationships that could be construed as a potential conflict of interest.
