# Supplementary material for: Histone Demethylases ELF6 and JMJ13 Antagonistically Regulate Self-Fertility in Arabidopsis
Source: Front Plant Sci. 2021 Feb 12;12:640135. doi: 10.3389/fpls.2021.640135 (PMC7907638; doi:10.3389/fpls.2021.640135)
Supplement: Supplementary file 2 [file Data_Sheet_1.DOCX]

Supplementary Material

# Supplementary Figures

**
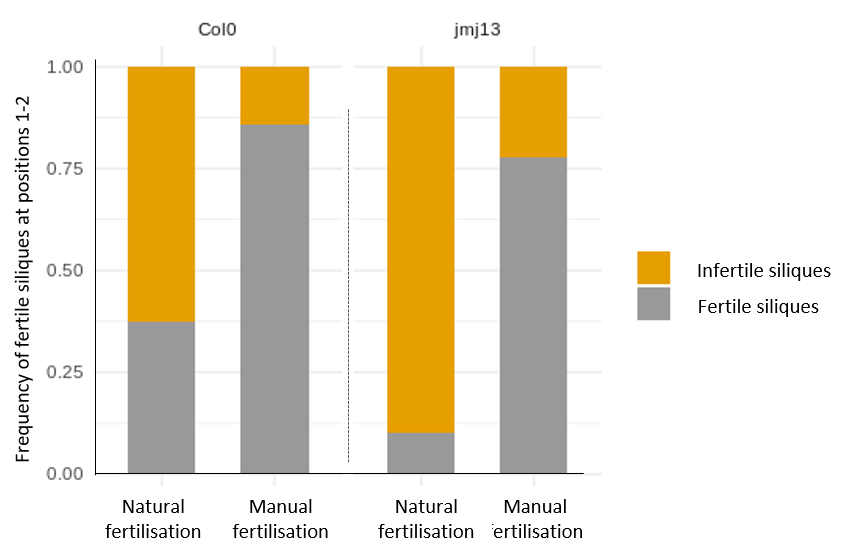
**

**Supplementary Figure 1.** Manual self-pollination was performed by removing the stamen from a particular flower and rubbing its anthers onto the stigma of the same flower. Performing this procedure for the first 2 flower positions of Col-0 and jmj13 plants restored their fertility (36 Col-0 flowers and 40 jmj13 flowers were assessed). The proportion of fertile flowers is shown in grey whereas the proportion of infertile flowers is shown in gold.


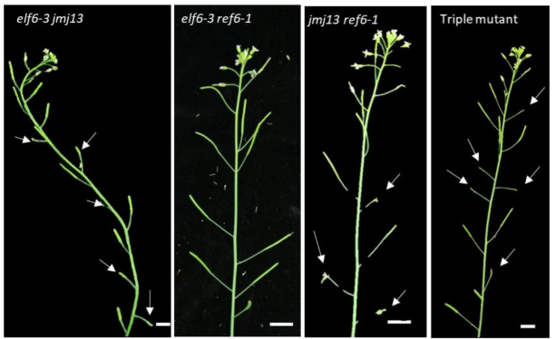


**Supplementary Figure 2.** Fertility phenotypes of H3K27me3 demethylase double and triple mutants. White arrows indicate aborted siliques caused by failed self-pollination. Scale bars represent 1cm.


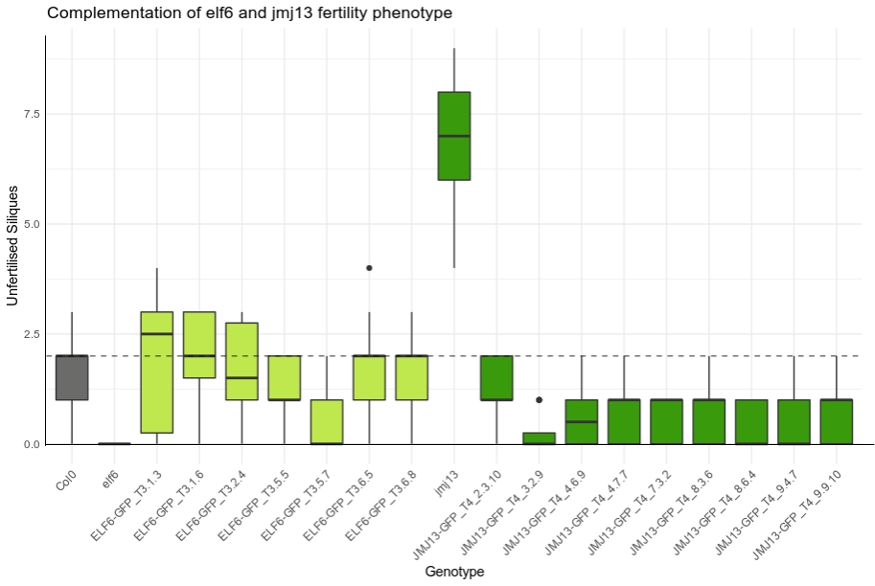


**Supplementary Figure 3.** Complementation of the elf6 and jmj13 fertility phenotypes by introduction of ELF6-GFP and JMJ13-GFP constructs into elf6 and jmj13 genetic backgrounds respectively. T2 offspring were screened by hygromycin and checked for a 75% resistance frequency to ensure only 1 copy of the construct was present. T3 lines were then screened and only the homozygous offspring (showing 100% hygromycin resistance) retained for characterisation of the fertility phenotype. The number of unfertilised siliques was counted up to the 10th silique for at least 8 mature primary inflorescences for each genotype. At least 5 plants were phenotyped for each transgenic line, and at least 10 plants were phenotyped for Col-0, elf6 and jmj13.


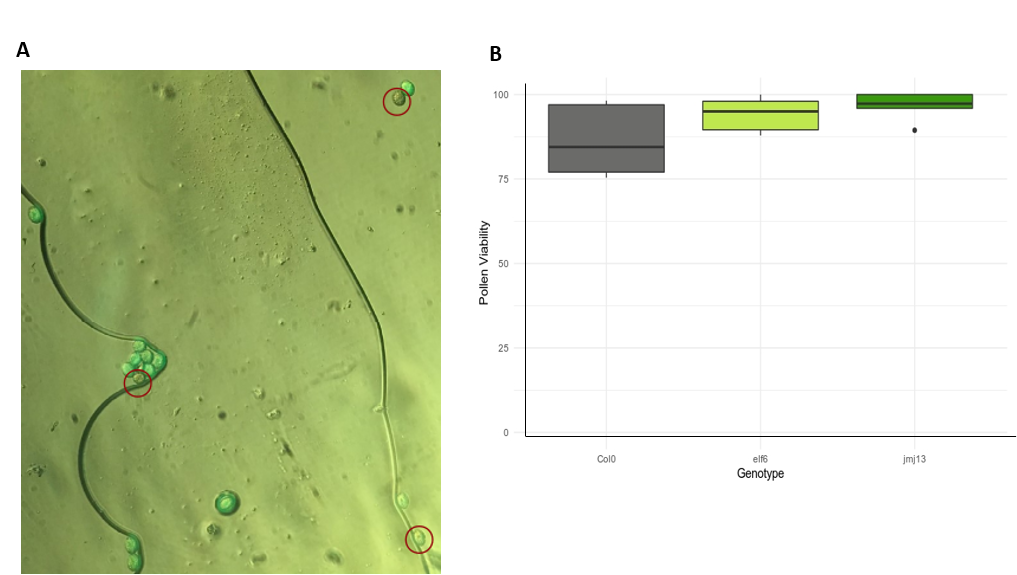


**Supplementary Figure 4.** Pollen viability of Col-0, and H3K27me3 demethylase single mutants. **A** Fluorescein diacetate stained pollen grains could be differentiated into live and dead cells by their fluorescence. In this image the dead pollen grains with low fluorescence are circled in red. **B** After scoring at least 200 pollen grains from 5 different flowers for each genotype, no significant difference was found between jmj13, elf6 and Col-0 (p > 0.05, t-test).


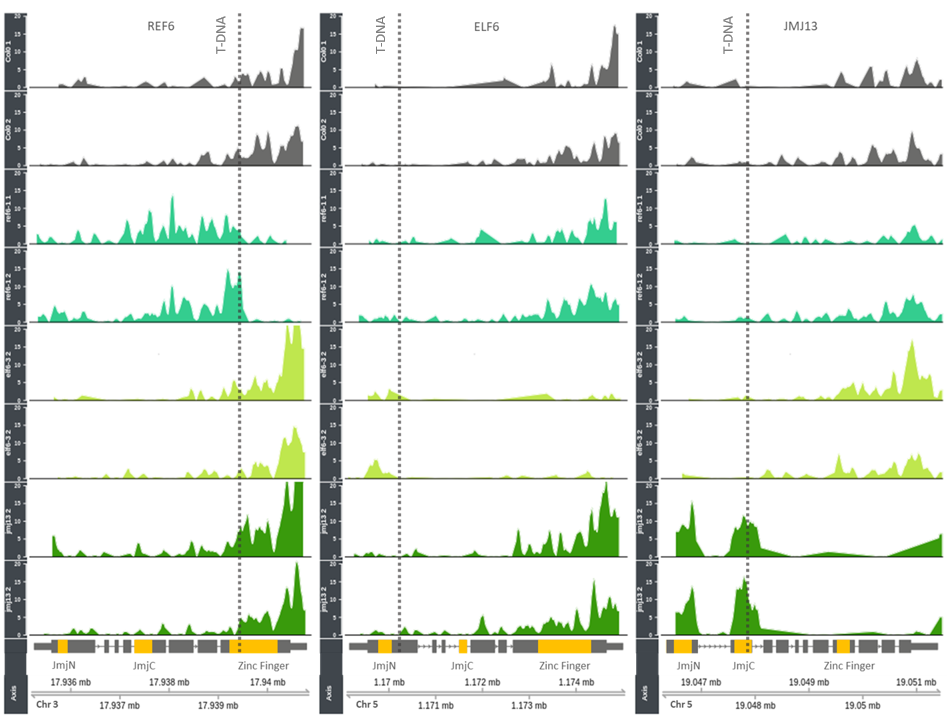


**Supplementary Figure 5.** Genome alignment of the demethylase mutant RNAseq reads allows us to observe the changes in transcriptome in higher detail. By zooming into the ref6, elf6 and jmj13 loci, it was observed that the REF6 T-DNA truncate is still be expressed to near wild-type levels. Below the read alignment, the gene model can be seen. The location of the T-DNA insertion is displayed as a dotted vertical line, and the JMJ and Zinc finger domains of each gene are highlighted in the gene model as yellow blocks. Genomic coordinates are plotted on the x-axis whilst the relative read alignment abundance is plotted on the y-axis.


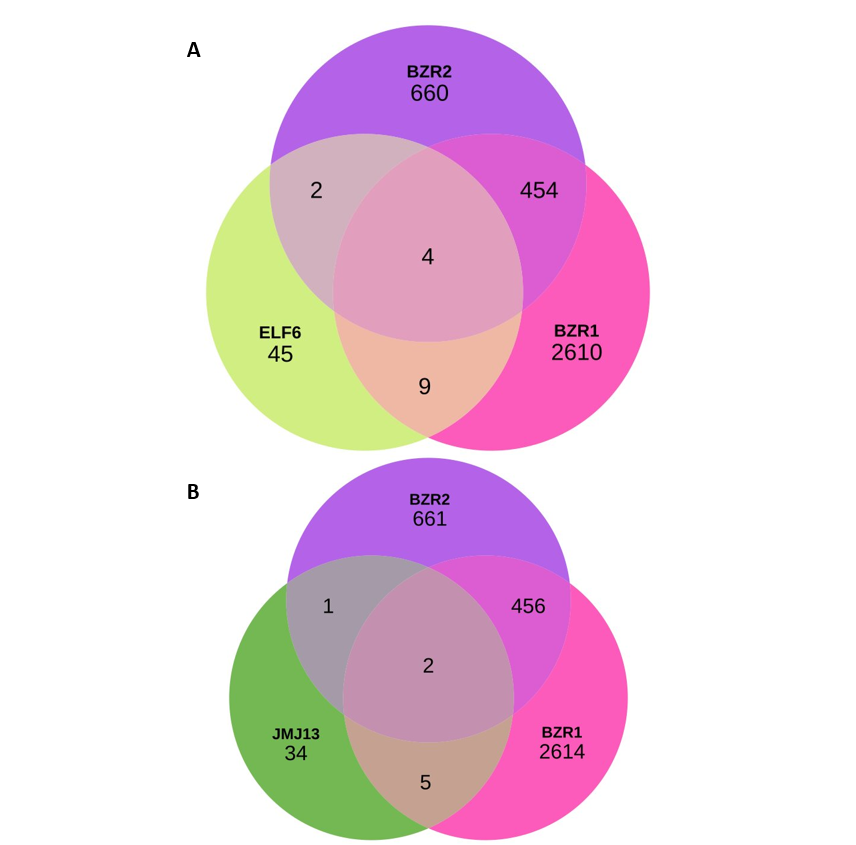


**Supplementary Figure 6.** Non-proportional venn diagrams depicting the overlap between the ChIP-seq validated target genes of BZR2 and BZR1 and the predicted target genes of ELF6 (A) and JMJ13 (B).


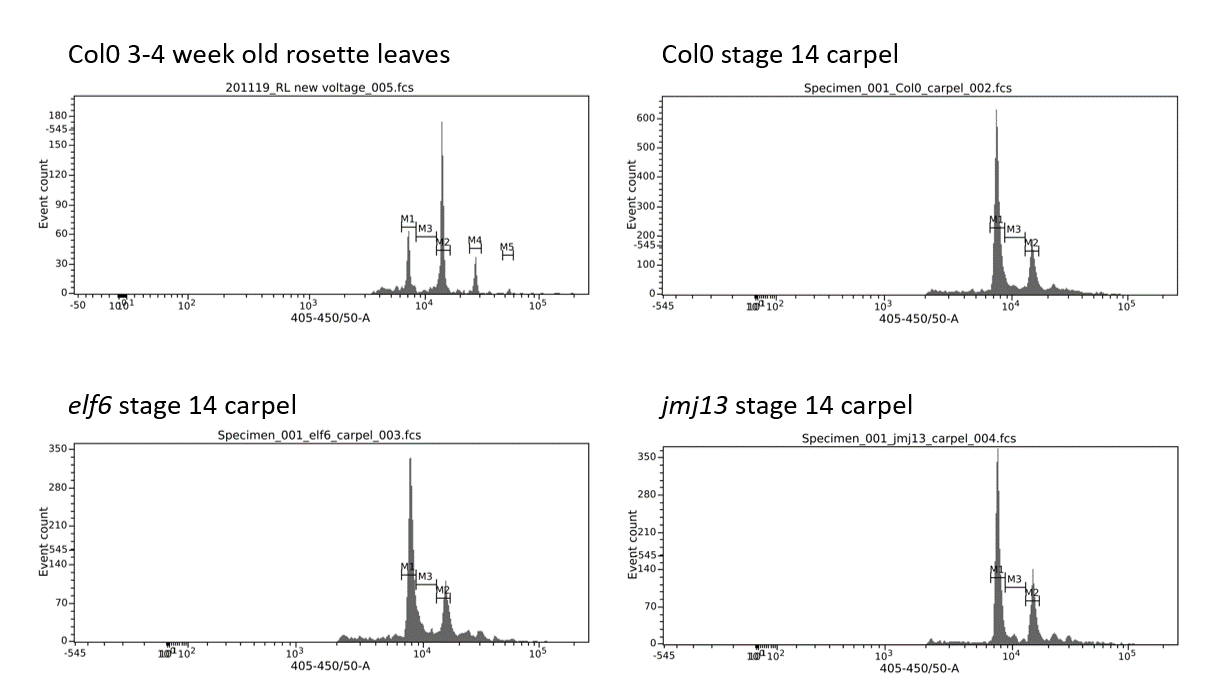


**Supplementary Figure 7.** Flow cytometry of DAPI stained nuclei revealed that no endoreduplication takes place in the carpels up to floral developmental stage 14, in contrast to rosette leaves where significant 8C and 16C peaks can be observed. It was also demonstrated that loss of elf6 and jmj13 had no effect on the degree of endoreduplication.


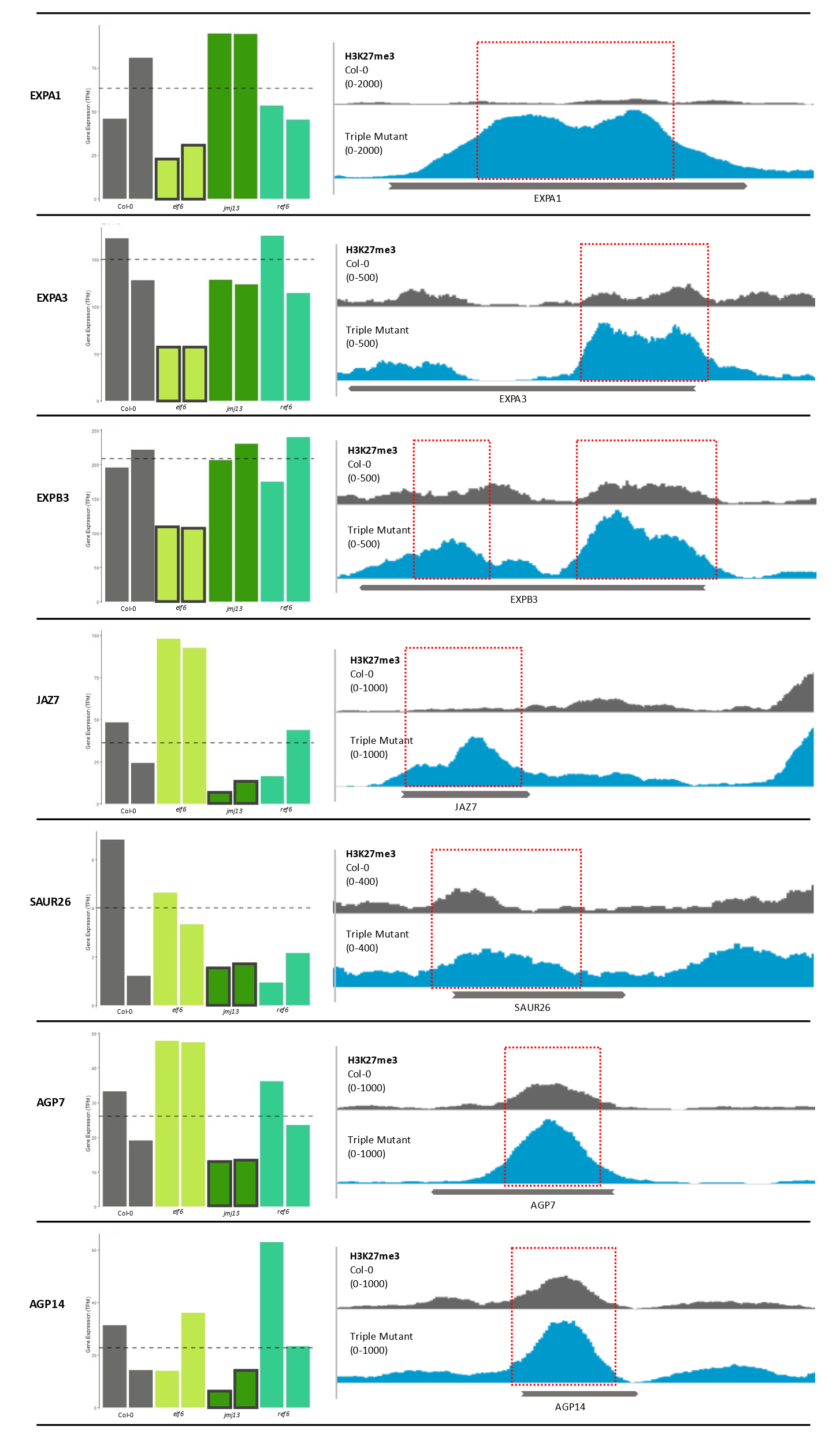


**Supplementary Figure 8.** Gene expression and H3K27me3 changes to ELF6 and JMJ13 target genes in demethylase knock-out mutant inflorescences. For each gene, the left panel shows the gene expression measured in transcripts per million (TPM), with a dashed line representing the mean TPM of the wildtype Col-0. A bold border on the gene expression bar chart highlights the expression levels of predicted target genes in the knockout mutants of the respective demethylases. The right-hand side panel depicts the H3K27me3 wiggle plot for each gene, where the y axis depicts ChIP-seq read coverage (re-analysis using raw data from Yan et al ., 2018; range detailed in parentheses next to each plot) and the x axis plotting genomic coordinates around the gene of interest. The gene model of the gene of interest is aligned to the genomic coordinates below the wiggle plot.
